# Experimental Study on Delivery Performance of an Automated Preloaded Intraocular Lens Injector System for Corneal and Sclerocorneal Incisions

**DOI:** 10.1155/2021/5548493

**Published:** 2021-03-31

**Authors:** Tetsuro Oshika, Noriyuki Sasaki

**Affiliations:** ^1^Department of Ophthalmology, Faculty of Medicine, University of Tsukuba, Ibaraki, Japan; ^2^Alcon Japan Ltd., Tokyo, Japan

## Abstract

**Purpose:**

To evaluate delivery performance of an automated preloaded intraocular lens (IOL) injector systems (AutonoMe) in the porcine eyes.

**Methods:**

In the freshly excised porcine eyes, lens removal and IOL implantation were performed. There were 4 groups (10 eyes per group) with different incision site and size: 2.2-mm and 2.4-mm corneal incisions and 2.2-mm and 2.4-mm sclerocorneal incisions. Delivery performance and wound enlargement of AutonoMe were analyzed and compared with those of iTec and iSert from a previous study.

**Results:**

There were a few minor troubles associated with AutonoMe, such as overriding plunger within cartridge and trapped trailing haptic during IOL insertion, but the incidence was low. Other interactions were not observed, such as IOL adherence to plunger, sudden ejection of IOL, intrawound lens manipulation, IOL behavior, and gross damage to IOL. AutonoMe caused significantly less wound enlargement for both corneal and sclerocorneal incisions than other injector devices. Wound enlargement by using AutonoMe was significantly smaller with 2.4-mm corneal incision than with 2.2-mm corneal incision, but the final incision size was still smaller with 2.2-mm corneal incision. For sclerocorneal incisions, the amount of wound stretch was not different between 2.2 and 2.4 mm incisions.

**Conclusion:**

The wound enlargement caused by the automated preloaded insertion system, AutonoMe, was smaller than that of other preloaded injectors for both corneal and sclerocorneal incisions. There were a few minor technical events during IOL insertion, but the overall incidence was low.

## 1. Introduction

There have been uninterrupted technological advancements in the field of cataract surgery to provide safer and more effective surgical treatments to patients. One of them is the advent of preloaded intraocular lens (IOL) delivery system. The merits of preloaded IOL delivery systems include prevention of IOL setting errors and potential IOL damages, elimination of variability in manual loading, reduced surgical time, fewer surgical instruments, decreased operative complexity and cost, and lower contamination risk of instruments with foreign bodies and/or microorganisms [1–5].

In most of IOL injectors, the plunger is pushed manually by either screw or push mechanisms. With the screw-type system, surgeons need to use both hands, one to hold the injector and the other to activate the screw, whilst the push-type injector system allows for single-hand maneuver, enabling the surgeon to use the second hand to stabilize the eye. The downside of this method is the substantial force required to compress and move the IOL in the cartridge, followed by a sudden decline of the required force as the bulkiest part of the IOL leaves the cartridge tip-internal incision opening. This can lead to an abrupt, explosive delivery of the lens into the eye, which can severely damage the intraocular tissues. To mitigate the risk of sudden ejection, some delivery devices are equipped with spring-controlled mechanisms to achieve smooth and controllable IOL delivery. Furthermore, a motorized injector system (AutoSert, Alcon Laboratories, Inc.) was developed to overcome these drawbacks of manual injectors [6–8]. This device enables the surgeons to stabilize the eye with the second hand, while insertion of the IOL is conducted at a controlled surgeon-determined speed. It was reported that the motorized injector causes less incision enlargement and preserves better wound architecture integrity than the manual injector [6–8]. This IOL delivery system, however, consists of a specialized nondisposable handpiece and a driving unit built in the phacoemulsification machine, and thus it is not possible to manufacture a disposable, preloaded-version of this system.

An automated preloaded IOL delivery system (AutonoMe, Alcon Laboratories, Inc.), which features the CO_2_-powered delivery mechanism, has been recently developed. The plunger is mechanically moved forward by pressing the speed control lever on the injector, with variable velocity according to the depression of the lever. When the surgeons lift the lever, the plunger stops advancement immediately. One clinical study [9] reported that this automated preloaded system yields less corneal tissue trauma compared with a manual injector system, and two experimental studies evaluated enlargement of corneal incision associated with the automated preloaded system [10, 11]. However, there has been no study on the influence of this new injector system on delivery performance and sclerocorneal wound enlargement. The present in vitro study was conducted to evaluate the delivery performance of the automated preloaded delivery system for IOL implantation via clear corneal and corneoscleral incisions in the cataract surgery model using the porcine cadaver eyes. Delivery performance was evaluated by assessing several operative parameters such as incision size as well as interactions between injectors and lenses that are indicatives of consistency and predictability of the injectors. Our previous study evaluated the performance of manual preloaded IOL injector systems, [5] while the current study aims to assess automated preloaded IOL injector system.

## 2. Materials and Methods

### 2.1. Preloaded Injector Systems

The automated preloaded delivery system (AutonoMe CNA0T0, hereafter referred to as system A) is a single-use IOL delivery system which contains hydrophobic acrylic foldable IOL (Clareon CNA0T0 IOL, Alcon Laboratories, Inc.). This device features a speed control lever interface for single-handed IOL delivery. The plunger is driven by compressed CO_2_ gas contained in the handpiece, and speed of IOL advancement is controlled by varying the lever depression. There is a depth guard at the tip of the nozzle. In the current study, we used IOLs of the same power (+21.0 diopters).

### 2.2. Porcine Eyes

This study used 40 freshly enucleated porcine whole eye globes from animals that were about 6 months of age and weighed 225 to 270 lb (105 to 125 kg). The eyes were purchased from Tokyo Shibaura Entrails Ltd. and stocked in a laboratory environment under controlled environment. The following criteria had to be met for the eyes to be used in this study: delivery to the laboratory within 1 day of preservation and having a noncloudy cornea, no vitreous leakage, and no apparent ocular damage. There were four study groups, with 10 eyes per group, with different incision site and size: 2.2-mm corneal incision, 2.4-mm corneal incision, 2.2-mm sclerocorneal incision, and 2.4-mm sclerocorneal incision. The order of surgery for 4 groups was randomly determined.

### 2.3. Surgical Procedures

All surgeries were performed by an experienced ophthalmic surgeon (T.O.) in the operating room with an ambient temperature of approximately 23°C. In brief, the porcine eye was incubated in an eye tissue holder at 37°C. A side-port incision was created using a disposable steel knife (Alcon Laboratories, Inc.) and the anterior chamber was filled with a dispersive ophthalmic viscosurgical device (OVD) (Viscoat, Alcon Laboratories, Inc., sodium hyaluronate 3.0%-chondroitin sulfate 4.0%); then, a corneal or corneoscleral incision was created using a disposable keratome (Alcon Laboratories, Inc.). The keratome size was selected according to the study protocol. Following capsulorhexis and hydrodissection, phacoemulsification and aspiration of the lens were performed with a phacoemulsification unit (Centurion Vision System with 0.9 mm 45-degree Intrepid Balanced tip and 0.9 mm Intrepid Ultra Infusion Sleeve, Alcon Laboratories, Inc.). The capsular bag and anterior chamber were filled with cohesive OVD (Provisc, Alcon Laboratories, Inc., sodium hyaluronate 1.0%), and the preimplantation incision size was measured with an incision gauge (AE-1582T, Crandall Capsulorhexis Incision Gauges: Set of 18 Titanium Blades, 1.5–3.2 mm, ASICO, LLC). The cartridge of the injector system was filled with a dispersive OVD (Viscoat, Alcon Laboratories, Inc.), and the IOL was implanted into the capsular bag. The postimplantation incision size was measured. The cartridge nozzle tip was inspected for any signs of fracture or splitting using a stereomicroscope at x32 magnification. These instrumentations and examination methods are identical to those employed in our previous study [5].

### 2.4. Examinations

Based on the visual observation and analysis of recorded surgical footages, we evaluated the testing parameters.  Table 1 lists the IOL delivery system interactions that were evaluated. The wound enlargement caused by IOL delivery was calculated by subtracting the pre-IOL implantation wound size from the post-IOL implantation wound size.

### 2.5. Statistical Analysis

A descriptive summary for each IOL delivery system interaction was provided. Statistical comparisons between study groups for each incision site (corneal and sclerocorneal) were performed using Fisher's exact test. Given the potentially correlated nature of intraocular measurements for delivery system interactions, a generalized estimating equation (GEE) was also planned. However, the computation algorithm did not converge due to low number of events and no solution was produced. For the data of wound enlargement and final wound size, *t*-test or one-way analysis of variance (ANOVA) was conducted for each incision site to assess the statistical significance. Because of the exploratory nature of this study, adjustments for multiplicity were not applied.

The delivery performance of system A in this study was compared with the data from a previous study using the same procedures with the same incision gauge as this study [5], which included Tecnis iTec preloaded injection system with Tecnis single-piece IOL (system iT) (Johnson & Johnson Vision Care, Inc.) (2.2-and 2.4-mm corneal incisions and 2.2- and 2.4-mm sclerocorneal incisions) and the Vivinex iSert preloaded injection system with Vivinex XY1 IOL (system iS) (Hoya Surgical Optics, Inc.) (2.0-mm corneal incision and 1.8-mm sclerocorneal incision). The incision size for each system was based on the specifications provided by the manufacturers. The diameter of nozzle tip of each device as reported by Nanavaty and Kubrak-Kisza [4] was 2.011 mm, 1.820 mm, and 1.684 mm with AutonoMe, iTec, and iSert, respectively.

## 3. Results

The IOL delivery system interactions of system A in this study were compared with those of other injectors in the previous study [5]. For corneal incisions, overriding plunger within cartridge was observed with system A 2.2 mm and 2.4 mm, and trapped trailing haptic occurred with system A 2.2 mm (Table 2), but the incidence of those observations was not significantly different among groups (*P* > 0.05). Incision enlargement ≥0.2 mm was seen with system A 2.2 mm, but not with system A 2.4 mm. Incision enlargement ≥0.2 mm was commonly found with all other injector systems, of which incidence was significantly difference among devices (*P* < 0.001), with the lowest occurrence (0/10) with system A 2.4 mm. Other interactions were not seen with system A 2.2 mm and 2.4 mm.

For sclerocorneal incision, incision enlargement ≥0.2 mm was noticed with all delivery systems (Table 3), and the incidence was significantly different among devices (*P* < 0.001), with lower occurrence with system A 2.2 mm and 2.4 mm than other systems. Other interactions were not observed with system A 2.2 mm and 2.4 mm.

**Figure 1:**
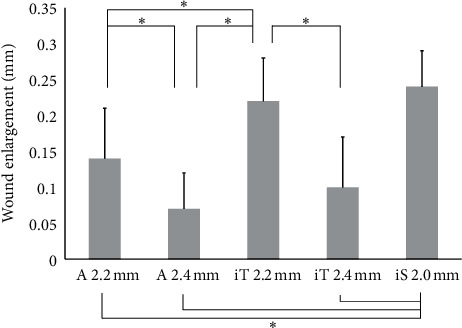
Mean enlargement with corneal incisions (±SD) (^*∗*^*P* < 0.05, one-way analysis of variance) (A = AutonoMe; iT = iTec; iS = iSert).

The amount of incision size enlargement caused by IOL implantation with system A was compared with that of other injectors. For corneal incisions (Figure 1; Table 4), system A 2.4 mm was associated with the smallest incision enlargement, which was significantly smaller than those of system A 2.2 mm, system iT 2.2 mm, and system iS 2.0 mm. With system A 2.2 mm, incision enlargement was significantly smaller than system iT 2.2 mm and iS 2. 0 mm.

**Figure 2:**
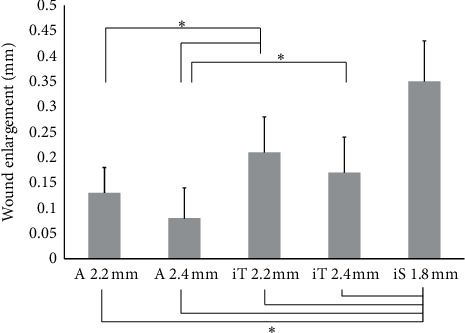
Mean enlargement with sclerocorneal incisions (±SD) (^*∗*^*P* < 0.05, one-way analysis of variance) (A = AutonoMe; iT = iTec; iS = iSert).

For sclerocorneal incision (Figure 2; Table 5), system A 2.4 mm showed the smallest enlargement, which was significantly smaller than those of system iT 2.2 mm, iT 2.4 mm, and iS 1.8 mm. The incision enlargement with system A 2.2 mm was significantly smaller than that of system iT 2.2 mm and iS 1.8 mm.

**Figure 3:**
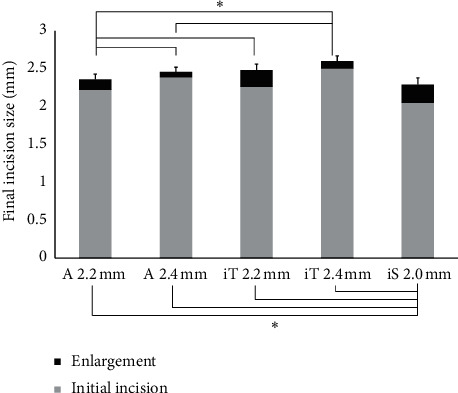
Mean final incision size (initial incision size + enlargement) with corneal incisions (±SD) (^*∗*^*P* < 0.05, one-way analysis of variance) (A = AutonoMe; iT = iTec; iS = iSert).

The final incision size measured after IOL implantation was compared among devices. For corneal incision (Figure 3; Table 4), the final incision with system A 2.2 mm was significantly smaller than that with system A 2.4 mm, system iT 2.2 mm, and system iT 2.4 mm, and significantly larger than that with system iS 2.0 mm. The final incision size with system A 2.4 mm was significantly smaller with that of system iT 2.4 mm, but significantly larger than that of system iS 2.0 mm.

**Figure 4:**
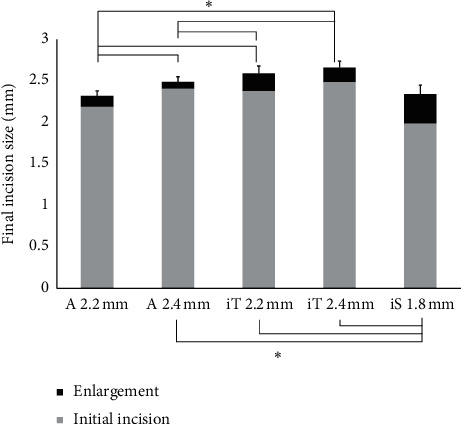
Mean final incision size (initial incision size + enlargement) with sclerocorneal incisions (±SD) (^*∗*^*P* < 0.05, one-way analysis of variance) (A = AutonoMe; iT = iTec; iS = iSert).

For sclerocorneal incision, system A 2.2 mm was associated with significantly smaller final incision size than system A 2.4 mm, system iT 2.2 mm, and system iT 2.4 mm (Figure 4; Table 5). The final incision size with system A 2.4 mm was significantly smaller than those with system iT 2.2 mm and system iT 2.4 mm, but significantly larger than that of system iS 1.8 mm.

## 4. Discussion

As for the IOL delivery performance of system A, automated preloaded IOL delivery system AutonoMe, we observed several minor troubles during IOL implantation procedures, such as overriding plunger within cartridge and trapped trailing haptic for corneal incisions (Table 2). These events, however, were not serious in severity and low in frequency. In addition, other interactions were not seen with system A, such as IOL adherence to plunger, sudden ejection of IOL, intrawound lens manipulation, IOL behavior, and gross damage to IOL (Tables 2 and 3). After implantation, there were a few cases of incision enlargement ≥0.2 mm with system A, but the incidence was significantly lower than other devices. Inspection under microscope magnification after IOL implantation revealed that splitting of the cartridge tip was not seen with system A, in contrast to 100% occurrence with system iS for both corneal and sclerocorneal incisions. Wang et al. [12] also conducted experimental delivery performance assessment of several IOL injectors, including UltraSert with a depth-guard nozzle tip (Alcon Laboratories, Inc.), iTec, and iSert, and reported the following findings; IOL adherence to the delivery system plunger tip was frequently observed with system iT; a trapped trailing haptic during IOL delivery was seen with systems iT and iS; and crack was found in all iS systems at the corner of the tip lip, resulting in a split in the nozzle tip. Mendicute et al. [13] clinically assessed the performance of preloaded IOL delivery systems, including UltraSert, iTec, and iSert. They found that trapped trailing haptic was frequently associated with iTec, nozzle tip splitting and plunger tip adherence were seen with iSert, and none of these occurred with UltraSert. Ong et al. [1] conducted clinical evaluation of AcrySert preloaded IOL injector system (Alcon Laboratories, Inc.) and demonstrated that there were several cases of haptic-optic adhesion, overriding of the plunger above the optic, trapped trailing haptic, and damage of optic edge. Judging from the current and previous findings, it seems that AutonoMe is associated with a low risk of intraoperative complications attributable to IOL delivery system manipulations.

In the present study, most of the IOL delivery system interactions were minor. To deal with the interactions, simple maneuvers were added, which required slightly prolonged operation time. If they occur during surgery, clinically significant sequences would not be anticipated. An in vitro study, however, indicated that haptic break caused by delayed trailing haptic as well as intrawound manipulation can occur with preloaded IOL delivery systems [5]. Clinically, a case of fractured optic during IOL implantation using a preloaded delivery system was reported [14]. Even though these are rare complications, further refinement is needed to sophisticate IOL preloaded delivery systems.

Some extent of incision enlargement and stretch pertain to intraocular surgery, especially when IOL injector systems are used [6, 7, 13, 15–17]. Wound enlargement can occur at various steps of cataract surgery, but mostly during lens insertion procedures [18]. In our study, incision size enlargement by IOL insertion was seen with all injector devices, and the degree of enlargement differed significantly among groups. Generally, a smaller preimplantation incision resulted in more enlargement than a larger preimplantation incision when the same IOL injector was employed. Significantly greater stretch was induced by system A 2.2 mm than system A 2.4 mm, and system iT 2.2 mm caused significantly larger enlargement than system iT 2.4 mm. Kleinmann and Kleinmann [19] utilized a finite element model and demonstrated that the stress induced on 2.2-mm incision margins was about 9% greater compared to the stress induced on 2.4-mm incision margins. It can be naturally assumed that implantation of IOL through a tighter incision will induce greater stress on incision structures. Enhanced stress on the corneal wound margins could lead to uncontrolled micro or even macro rips of the wound margins, unlike the controlled wounds created using an incision knife [19]. These would significantly jeopardize the integrity of cataract surgery wounds. Thus, it is important to select proper wound size that matches the IOL delivery system.

As shown in the results, incision size enlargement was smaller with system A than with system iT and iS. Liu et al. [10] similarly reported that AutonoMe preloaded delivery system provided smaller corneal incision enlargement compared with other systems, such as UltraSert, Vivinex iSert, and Tecnis iTec. Cennamo et al. [11] demonstrated that AutonoMe preloaded injector ensured less trauma to the wound and contributed to preserving the endothelial side of the incision. There are at least four possible explanations for these results. First, the use of mechanical force to drive the plunger may have led to less damage to the wound. Several studied evaluated the influence of AutoSert, a motorized IOL injector system, on the integrity of cataract surgery incision. An in vitro morphological study using pig eyes by Mencucci et al. [8] demonstrated that AutoSert provided more regular and less damaged incisions than the manual injectors, especially after high-power IOL implantation. Khokhar et al. [7] found that AutoSert caused significantly less incision enlargement and better preserved wound integrity in a clinical study. Allen et al. [6] showed that the motorized injector induced less wound enlargement, especially when used with a faster speed. It was speculated that the more rapidly the IOL is injected through the wound, the less time is needed for the IOL to start to re-expand (in the wound) toward its original shape once it leaves the cartridge opening. They also postulated that the difference between the motorized injector and the manual injector is due to a combination of factors, such as the different pressure required to maintain close apposition of the cartridge tip to the wound, discontinuous movement of the IOL through the wound with the manual injector, rotation of the tip around the *z*-axis of the injector when turning the screw, and yaw (side-to-side movement) in relation to the wound entrance when manipulating the manual injector [6]. Mastropasqua et al. [9] demonstrated that the AutonoMe, CO_2_-powerd automated injector, caused less tissue damage to the cornea compared with the Monarch III injector, and this result is probably attributable to a greater amount of corneal stress and trauma at the wound provided by the conventional injector system compared to the automated delivery system. It was postulated that automated delivery system prevents surgeons' inadvertent maneuvers during IOL implantation as it could occur when using manual injectors, and can standardize the injection procedures [9].

Second, presence of the depth-guard at the noddle tip may have contributed to the results. The depth guard is designed to prevent the excessive penetration of the nozzle tip into the tunnel [12]. Since inadvertently deep insertion of the cartridge nozzle can lead to significant stretch and enlargement of the tissue, it appears that the depth guard contributes to the maintenance of wound size during IOL implantation by limiting too deep insertion [5, 9, 12]. Negishi et al. [20] reported that AutonoMe automated delivery system with the depth guard enabled safe IOL implantation through a 2.4-mm corneal incision. In addition, the presence of depth guard seems to enhance the mechanical strength of the nozzle, which makes the cartridge more resistant to splitting and stretching by suppressing volume increases.

Third, differences in insertion techniques should be considered. In this study, system A and iT utilized the wound-assisted (docking) method, while system iS employed the cartridge-insertion (direct) method. A clinical analysis [21] indicated that the final wound size and the changes in wound size by IOL insertion were larger with the direct method than with the docking method. The corneal hysteresis and resistance factors were also evaluated, but corneal biomechanical properties did not correlate with the final wound size. Kohnen and Klaproth [15] showed that use of an proper docking implantation technique could decrease the wound size for acrylic foldable IOL implantation. An in vitro study indicated that the IOL delivery system using the cartridge-insertion technique was associated with greater incision stretch than the IOL injectors used with the wound-assisted technique [5]. Thus, it is important to note that IOL insertion techniques exert substantial impacts on the integrity of cataract surgery wound.

Fourth, morphological investigation with scanning electron microscope revealed that the Monarch D cartridge was associated with more irregularities of the edge and external wall of the nozzle tip compared with the AutonoMe cartridge [9]. Roughness analysis with the atomic force microscope revealed that the Monarch D cartridge had greater roughness than AutonoMe cartridge [9]. The smoothness of the nozzle tip probably decreased the friction of the cartridge tip inside the corneal stroma and thus prevented microtraumatic events at the tunnel site.

The present study has several limitations. First, the size of keratome was selected according to the delivery system instruction or specification provided by the manufacturers. We are not certain whether the keratome size employed in this study was most appropriate for each IOL injector system; if a larger keratome was used to create the initial wound, incision enlargement could be smaller than what was observed in the current results. Particularly, only one knife size was used for the evaluation of iS with corneal and sclerocorneal incisions. Different results could have been obtained if system iS was tested through a less tighter wound. Second, the current study was conducted in a cataract surgery model using the porcine eyes. The living human eyes are different from the enucleated porcine eyes in various aspects. Although stretched tissues in living human tissues will eventually contract to some extent due to elasticity, it is unlikely that the porcine eyes shrink once stretched by an external force. Third, statistical results about the comparative effectiveness of system A with control injectors were not based on randomized, but cross-study comparisons. Fourth, each delivery system was tested in only 10 eyes, which is not enough to estimate the actual rate of complications encountered during implantation process. For that purpose, clinical studies involving a large cohort will be needed.

In conclusion, the current in vitro experiments indicated that system A, automated IOL delivery system AutonoMe, was associated with a few minor technical problems, such as overriding plunger within cartridge and trapped trailing haptic during IOL insertion, but overall incidence of IOL delivery system interactions was not high. System A caused the least amount of wound enlargement by IOL implantation for corneal and sclerocorneal incisions compared with other injector systems, resulting in acceptable final wound size.

## Figures and Tables

**Table 1:** Delivery device outcomes.

1	Intraocular lens adherence to delivery system plunger
2	Sudden ejection
3	Overriding plunger within cartridge
4	Intrawound lens manipulation
5	Trapped trailing haptic
6	Intraocular lens behavior
7	Gross damage to intraocular lens
8	Incision enlargement ≥0.2 mm after intraocular lens delivery
9	Cartridge tip splitting

**Table 2:** Occurrence of IOL delivery system interactions for corneal incisions.

Delivery system and knife size (mm)	IOL adherence to plunger	Sudden ejection of IOL	Overriding plunger within cartridge	Intrawound lens manipulation	Trapped trailing haptic	IOL behavior	Gross damage to IOL	Incision enlargement ≥0.2 mm	Cartridge tip splitting
A 2.2	0/10	0/10	1/10	0/10	1/10	0/10	0/10	5/10	0/10
A 2.4	0/10	0/10	1/10	0/10	0/10	0/10	0/10	0/10	0/10
iT 2.2	0/10	0/10	0/10	3/10	0/10	0/10	0/10	9/10	0/10
iT 2.4	1/10	0/10	0/10	3/10	0/10	0/10	0/10	2/10	0/10
iS 2.0	0/10	0/10	0/10	0/10	0/10	0/10	0/10	10/10	10/10

IOL = intraocular lens; A = AutonoMe; iT = iTec; iS = iSert.

**Table 3:** Occurrence of IOL delivery system interactions for sclerocorneal incisions.

Delivery system and knife size (mm)	IOL adherence to plunger	Sudden ejection of IOL	Overriding plunger within cartridge	Intrawound lens manipulation	Trapped trailing haptic	IOL behavior	Gross damage to IOL	Incision enlargement ≥0.2 mm	Cartridge tip splitting
A 2.2	0/10	0/10	0/10	0/10	0/10	0/10	0/10	3/10	0/10
A 2.4	0/10	0/10	0/10	0/10	0/10	0/10	0/10	1/10	0/10
iT 2.2	0/10	0/10	0/10	0/10	0/10	0/10	0/10	8/10	0/10
iT 2.4	0/10	0/10	0/10	0/10	0/10	0/10	0/10	8/10	0/10
iS 1.8	0/10	0/10	0/10	1/10	0/10	0/10	0/10	10/10	10/10

IOL = intraocular lens; A = AutonoMe; iT = iTec; iS = iSert.

**Table 4:** Mean enlargement and final size of corneal incisions.

Device (mm)	A 2.2 mm	A 2.4 mm	iT 2.2 mm	iT 2.4 mm	iS 2.0 mm
Mean incision enlargement	0.14 ± 0.07	0.07 ± 0.05	0.22 ± 0.06	0.10 ± 0.07	0.24 ± 0.05
Mean final incision size	2.36 ± 0.07	2.48 ± 0.06	2.48 ± 0.08	2.60 ± 0.07	2.29 ± 0.09

A = AutonoMe; iT = iTec; iS = iSert. Data are represented as means ± SD.

**Table 5:** Mean enlargement and final size of sclerocorneal incisions.

Device	A 2.2 mm	A 2.4 mm	iT 2.2 mm	iT 2.4 mm	iS 1.8 mm
Mean incision enlargement (mm)	0.13 ± 0.05	0.08 ± 0.06	0.21 ± 0.07	0.17 ± 0.07	0.35 ± 0.08
Mean final incision size (mm)	2.32 ± 0.06	2.49 ± 0.03	2.59 ± 0.09	2.66 ± 0.08	2.34 ± 0.11

A = AutonoMe; iT = iTec; iS = iSert. Data are represented as means ± SD.

## Data Availability

The datasets analyzed during the current study are available from the corresponding author upon request.

## Abbreviations

IOL: Intraocular lens.
